# Mitochondrial Heat Shock Response Induced by Ectromelia Virus is Accompanied by Reduced Apoptotic Potential in Murine L929 Fibroblasts

**DOI:** 10.1007/s00005-019-00554-5

**Published:** 2019-07-19

**Authors:** Zbigniew Wyżewski, Karolina P. Gregorczyk-Zboroch, Matylda B. Mielcarska, Magdalena Bossowska-Nowicka, Justyna Struzik, Joanna Szczepanowska, Felix N. Toka, Marek G. Niemiałtowski, Lidia Szulc-Dąbrowska

**Affiliations:** 1 Department of Preclinical Sciences, Faculty of Veterinary Medicine, Warsaw University of Life Sciences-SGGW, Ciszewskiego 8, 02-786 Warsaw, Poland; 2 Present Address: Department of Biochemistry, Faculty of Agriculture and Biology, Warsaw University of Life Sciences-SGGW, Warsaw, Poland; 3 Laboratory of Bioenergetics and Biomembranes, Department of Biochemistry, Nencki Institute of Experimental Biology, Polish Academy of Sciences, Warsaw, Poland; 4 Center for Integrative Mammalian Research, Ross University School of Veterinary Medicine, Basseterre, West Indies Saint Kitts and Nevis

**Keywords:** ECTV, Hsp60, Hsp10, Apoptosis, Mitochondria

## Abstract

Poxviruses utilize multiple strategies to prevent activation of extrinsic and intrinsic apoptotic pathways for successful replication. Mitochondrial heat shock proteins (mtHsps), especially Hsp60 and its cofactor Hsp10, are engaged in apoptosis regulation; however, until now, the influence of poxviruses on mtHsps has never been studied. We used highly infectious Moscow strain of ectromelia virus (ECTV) to investigate the mitochondrial heat shock response and apoptotic potential in permissive L929 fibroblasts. Our results show that ECTV-infected cells exhibit mostly mitochondrial localization of Hsp60 and Hsp10, and show overexpression of both proteins during later stages of infection. ECTV infection has only moderate effect on the electron transport chain subunit expression. Moreover, increase of mtHsp amounts is accompanied by lack of apoptosis, and confirmed by reduced level of pro-apoptotic Bax protein and elevated levels of anti-apoptotic Bcl-2 and Bcl-xL proteins. Taken together, we show a positive relationship between increased levels of Hsp60 and Hsp10 and decreased apoptotic potential of L929 fibroblasts, and further hypothesize that Hsp60 and/or its cofactor play important roles in maintaining protein homeostasis in mitochondria for promotion of cell survival allowing efficient replication of ECTV.

## Introduction

Apoptosis of the infected cell is an event that leads to disruption of the viral replication cycle and, consequently, to elimination of the infectious agent from the host tissues. Therefore, programmed cell death serves as a mechanism of unspecific antiviral immune response. For this reason, many viruses delay apoptosis to preserve infected cells as a replication platform and to facilitate further dissemination (Gregorczyk et al. 2014a; Mehta et al. 2015). Anti-apoptotic effects play an important role in the pathogenesis of various viral diseases, and contribute to persistence of infection or development of virus-dependent tumorigenesis (Henderson et al. 1991; Lima et al. 2008; Oh et al. 2010; Yuan et al. 2012).

Ectromelia virus (ECTV) is a member of the *Orthopoxvirus* genus of the *Poxviridae* family and is the causative agent of mousepox—a lethal disease of certain strains of mice (Esteban and Buller 2012). The *Poxviridae* family contains DNA viruses that are classified into two subfamilies: *Chordopoxvirinae* and *Entomopoxvirinae*, which contain agents that cause infections in vertebrates and invertebrates, respectively. From the epidemiological point of view, the most important genus within the *Chordopoxvirinae* subfamily is *Orthopoxvirus*, which contains viruses that cause systemic diseases. The most dangerous orthopoxvirus is variola virus (VARV), which is the etiological agent of smallpox, the disease that decimated human populations over hundreds of years all over the world (Fenner 2000). Global eradication campaign against smallpox was a spectacular success, and in 1980, the World Health Organization (WHO) officially declared that smallpox was eradicated. The last terminal case of the illness took place in Great Britain, in 1978. Exaggerated optimism, as a result of successful vaccine program, has caused premature discontinuation of vaccination and consequent decrease in herd immunity against orthopoxvirus. VARV stocks have not been destroyed and are still stored at two laboratories collaborating with WHO, in USA (Atlanta) and in Russia (Novosibirsk). Moreover, the Centers for Disease Control and Prevention in Atlanta considers smallpox as a category A bioterrorism agent, and therefore, an instrument of bioterrorism attacks or blackmails (Pennington 2003). Because experiments on VARV are generally prohibited (with the exception of the two laboratories mentioned earlier), ECTV is used extensively as a surrogate model to study pathogenesis of smallpox and other generalized orthopoxvirus infections (Buller 2004; Szulc-Dąbrowska et al. 2017).

The characteristic feature common for many representatives of the *Poxviridae* family is their anti-apoptotic ability (Nichols et al. 2017). Many poxviruses encode different types of virulence factors within their genomes that are able to interfere with extrinsic and/or intrinsic apoptotic pathways. Elimination of infected cells through apoptosis is a part of the host antiviral strategy and is an important process for controlling virus infection. Cell death implies disruption of viral replication cycle; therefore, anti-apoptotic activity of poxviruses may promote viral infection and disease progression (Gregorczyk et al. 2014a; Mehta et al. 2015).

Mitochondrial heat shock proteins (mtHsps)—Hsp60 and Hsp10, preferentially localized in the mitochondrial matrix—regulate apoptotic cell death (Ghosh et al. 2008; Gupta and Knowlton 2005; Shan et al. 2003). Hsp60 mediates correct folding of newly synthesized, as well as stress-denatured proteins, and in association with Hsp10 controls folding of proteins that are transported from the cytoplasm to the mitochondrial matrix (Parnas et al. 2012; Wyżewski et al. 2014). Hsp60 has both pro- and anti-apoptotic abilities; however, it is known mainly as a protein that counteracts apoptosis. Previous studies have suggested that the activity of Hsp60 may cause changes in the proportion of pro- and anti-apoptotic B-cell lymphoma-2 (Bcl-2) protein family members in a way that favors the latter. Hsp60 can contribute to decrease in pro-apoptotic Bcl-2-associated X protein (Bax) level and increase intracellular level of anti-apoptotic Bcl-2 and B-cell lymphoma-extra-large (Bcl-xL). Hsp60, as well as Hsp10, can modulate post-translational modification of Bcl-xL because they inhibit its ubiquitination allowing it to avoid degradation in the proteasome (Shan et al. 2003). Moreover, Hsp60 may counteract induction of intrinsic apoptotic pathway by performing mitoprotective functions. Due to chaperone activity, Hsp60, alone or in cooperation with Hsp10, is engaged in maintaining mitochondrial integrity and functionality. It has been shown that Hsp60 and its cofactor promote ATP synthesis, increase the activity of complex III and complex IV of the respiratory electron transport chain (ETC), as well as prevent cytochrome c release to the cytosol (Kleinridders et al. 2013; Lin et al. 2001).

Hsp60 has been found to regulate development and progression of infections caused by numerous viruses, including hepatitis B virus, human immunodeficiency virus, and influenza A virus (Wyżewski et al. 2018). However, the role of mtHsp in maintaining cell viability during orthopoxviral infection has not been elucidated. The regulatory role of Hsp60 in apoptosis supports the hypothesis that this chaperone may affect effectiveness of viral replication (Cohen-Sfady et al. 2009; Ghosh et al. 2008; Resa-Infante et al. 2011).

Previous studies have shown that ECTV infection increases intracellular level of chaperone proteins, including Hsp27, Hsp70, and Hsp90 in murine macrophages. Those proteins are able to inhibit both extrinsic and intrinsic apoptotic pathways (Cymerys et al. 2009). Recently, Hsp70 isoform, Hsp70 member 1B, was shown to be required for efficient ECTV replication (Cheng et al. 2018). However, the influence of orthopoxvirus infection on Hsp60 and its cofactor—Hsp10—has not been examined before. We, therefore, studied the impact of ECTV infection on the intracellular distribution of Hsp60 and Hsp10, as well as the levels of Hsp60, Hsp10, and chosen pro- and anti-apoptotic proteins from the Bcl-2 family. Our results showed that Hsp60 and Hsp10 localization remained mainly mitochondrial, while the expression of these proteins increased at later stages of infection. The increased expression of mtHsps was accompanied by decreased or increased levels of pro-apoptotic Bax or anti-apoptotic Bcl-2 and Bcl-xL proteins, respectively. Moreover, ECTV did not induce apoptosis in L929 fibroblasts during its 24 h replication cycle. Taken together, our results suggest that retained mitochondrial location and increased levels of Hsp60 and Hsp10 counteract mitochondria-induced apoptosis in murine fibroblasts during later stages of ECTV infection.

## Materials and Methods

### Cell Culture

L929 fibroblasts of H-2^k^ haplotype (ATCC CCL-1; LGC Standards, Teddington, UK) were cultured in Dulbecco’s Modified Eagle’s Medium supplemented with 4.5 g/L glucose (HyClone, Logan, UT, USA), 5% fetal bovine serum (FBS; Sigma-Aldrich, St. Louis, MO, USA), and 1% antibiotic/antimycotic solution (10,000 U/mL penicillin, 10 mg/ml streptomycin; 25 µg/ml amphotericin B; Sigma-Aldrich).

### Virus

Moscow strain of ECTV (ATCC 1374) was used in all experiments. ECTV was propagated and titrated by plaque-forming unit method in Vero (Green Monkey kidney epithelium) cell line (ATCC CCL-81). Virus stocks were stored in aliquots at − 70 °C until used. L929 cells were infected with ECTV at a multiplicity of infection of 5. After 1 h of virus adsorption, the cells were incubated in fresh culture medium enriched with 1% FBS at 37 °C in a humidified 5% CO_2_ atmosphere and harvested at 4, 8, 12, 18, and/or 24 h post-infection (hpi) for further experiments. Control cultures were identically processed, but not infected with ECTV.

### Immunofluorescence Staining for Microscopy

Immunofluorescence staining was performed, as previously described (Szulc-Dąbrowska et al. 2016), with minor modifications. Briefly, L929 fibroblasts were seeded on cover slips in a 24-well plate and infected with ECTV as described earlier. At 4, 8, 12, 18, and/or 24 hpi, the cells were fixed with 4% paraformaldehyde (PFA; Sigma-Aldrich) for 15 min. In some experiments, fixation was preceded by mitochondrial staining with 300 nm MitoRed (Sigma-Aldrich) for 20 min at 37 °C in a humidified 5% CO_2_ atmosphere. After fixation, cells were permeabilized with 0.5% Triton X-100 (Sigma-Aldrich) in Dulbecco’s Phosphate-Buffered Saline (DPBS) and blocked with 2% bovine serum albumin (BSA; Sigma-Aldrich) in 0.1% Triton X-100 in DPBS. Slides were stained for 1 h with the following primary antibodies (Abs): mouse monoclonal antibody (mAb) anti-Hsp60 [3G8] (1:4000; Thermo Fisher Scientific, Waltham, MA, USA) and rabbit mAb anti-Hsp10 [EPR4476] (1:50; Abcam, Cambridge, UK). Next, the cells were stained for 45 min with the following secondary Abs: donkey anti-mouse Ab conjugated with fluorescein isothiocyanate (FITC) or rhodamine Red X (1:50; Jackson ImmunoResearch Laboratories, Inc., West Grove, PA, USA) or the donkey anti-rabbit Ab conjugated with FITC or rhodamine Red X (1:50; Jackson ImmunoResearch Laboratories, Inc.). Viral antigens were labeled with rabbit Abs anti-ECTV conjugated with FITC (1:200) for 60 min. Nuclear and viral DNA was stained with Hoechst 33342 (Sigma-Aldrich) for 5 min. After staining, slides were mounted using ProLong Gold Antifade Reagent (Invitrogen Life Technologies, Carlsbad, CA, USA).

### Confocal and Fluorescence Microscopy

Slides were examined using confocal microscopes Leica SP8 SMD (Leica Microsystems, Wetzlar, Germany) and Zeiss LSM780 (Carl Zeiss, Oberkochen, Germany), and fluorescence microscope Olympus BX60 equipped with Color View cooled CCD camera and Cell^F software (Olympus, Tokyo, Japan). The images were analyzed with the use of ImageJ software (NIH, Bethesda, MD, USA).

### Mitochondrial Mass Measurement by Flow Cytometry

Mitochondrial mass of L929 fibroblasts was measured using Mitotracker Green FM fluorescent dye (Thermo Fisher Scientific). At 4, 8, and 18 hpi, cells were incubated with 300 nm MitoTracker Green FM for 20 min at 37 °C. Next, the cells were washed and suspended in DPBS and analyzed using BD LSR Fortessa^TM^ flow cytometer and BD FACSDiva 7.0 software. Gating of the cells was based on the FSC/SSC characteristics. The relative mitochondrial mass was determined by measuring mean fluorescent intensity (MFI) in FITC emission spectra for 10,000 cells and subsequently comparing MFI in test and control samples.

### Measurement of Intracellular Protein Level by Flow Cytometry

Control and ECTV-infected L929 fibroblasts at 4, 8, 12, 18, or 24 hpi were fixed with 4% PFA for 15 min and permeabilized with 0.1% Triton X-100 in DPBS. After blocking with 2% BSA in 0.1% Triton X-100 in DPBS, cells were stained for 1 h with the following primary Abs: rabbit anti-Bcl-2 pAbs (1:100; Thermo Fisher Scientific) and mouse anti-Bcl-x [4/Bcl-x] mAbs (1:50; BD Biosciences). Next, the cells were stained for 45 min with donkey anti-mouse Abs conjugated with allophycocyanine (APC; 1:200; Jackson ImmunoResearch Laboratories, Inc.), or donkey anti-rabbit Abs conjugated with FITC or rhodamine Red X (1:200; Jackson ImmunoResearch Laboratories, Inc.). The cells were analyzed using flow cytometry. Gating of the cells was based on the FSC/SSC profile. The relative level of proteins was determined by measuring the MFI for 10,000 cells and subsequently by comparing MFI in test and control samples.

### Western Blotting

L929 cells were lysed using RIPA Lysis and Extraction Buffer (Thermo Fisher Scientific) supplemented with 1% solution of protease and phosphatase inhibitors (Thermo Fisher Scientific). The protein concentration in lysates was determined using MicroBCA Protein Assay Kit (BCA; Thermo Fisher Scientific), according to the manufacturer’s instructions. Sodium dodecyl sulfate polyacrylamide gel electrophoresis was performed to separate proteins. 10% polyacrylamide gels were used to resolve Hsp60, Bcl-2, Bcl-xL, Bax, caspase-3, and chosen subunits of ETC and ATPase, whereas 15% polyacrylamide gel was used to separate Hsp10. Electrophoresis was performed at 150 V for 1 h. Separated proteins were transferred onto polyvinylidene fluoride membranes at 100 V for 1 h. Next, membranes were incubated in 5% non-fat dry milk solution in 0.1% Tween 20 (Sigma-Aldrich) in DPBS to block unspecified binding sites. The blocked membranes were incubated at 4 °C overnight with the following primary Abs: mouse anti-Hsp60 mAb [3G8] (1:1000), rabbit anti-Hsp10 mAb [EPR4476] (1:10,000), mouse anti-Bcl-x mAb [4/Bcl-x] (1:500), rabbit anti-Bcl-2 pAb (1:1000), rabbit anti-Bax pAb (1:500; Abcam), mouse anti-GAPDH mAb [GA1R] (1:1000; Thermo Scientific, GAPDH served as protein loading control), and Total OXPHOS Rodent WB Antibody Cocktail (1:250; Abcam), consisting of mouse anti-NDUFB8 mAb [20E9DH10C12], mouse anti-SDHB mAb [21A11AE7], mouse anti-UQCRC2 mAb [13G12AF12BB11], mouse anti-MTCO1 mAb [1D6E1A8], and mouse anti-ATP5A mAb [15H4C4]. After washing, membranes were incubated for 2 h with appropriate secondary Abs: goat anti-mouse or anti-rabbit conjugated with horseradish peroxidase (1:5000; Santa Cruz Biotechnology). The detection of membrane-bound proteins was performed either by chemiluminescence or colorimetry. In the first case, membranes were incubated for 5 min with Pierce™ ECL Plus Western Blotting Substrate (Thermo Fisher Scientific) and chemiluminescence detected by autoradiography (Kodak, Rochester, NY, USA). The chromogenic detection of proteins was performed using 1-Step™ TMB-Blotting Substrate Solution (Thermo Fisher Scientific), containing 3,3′,5,5′-tetramethylbenzidine (TMB) solution. The protein bands were analyzed densitometrically using ImageJ program (NIH, USA) and normalized against the intensity of GAPDH. Values obtained in control samples were expressed as 100% and values in test samples (infected cells) were expressed relative to the control.

### Statistical Analysis

All measurements were obtained from at least three independent experiments. The normality of distribution was determined using the Shapiro–Wilk test. Statistical analysis of obtained results was performed using Student’s *t* test for independent or dependent samples, Mann–Whitney *U* test, or Wilcoxon signed-rank test, when appropriate. The Pearson’s correlation test was used to evaluate the level of linear relationship between chosen values (variables).

## Results

### Distribution of Hsp60 in L929 Cells During ECTV Replication Cycle

**Fig. 1:**
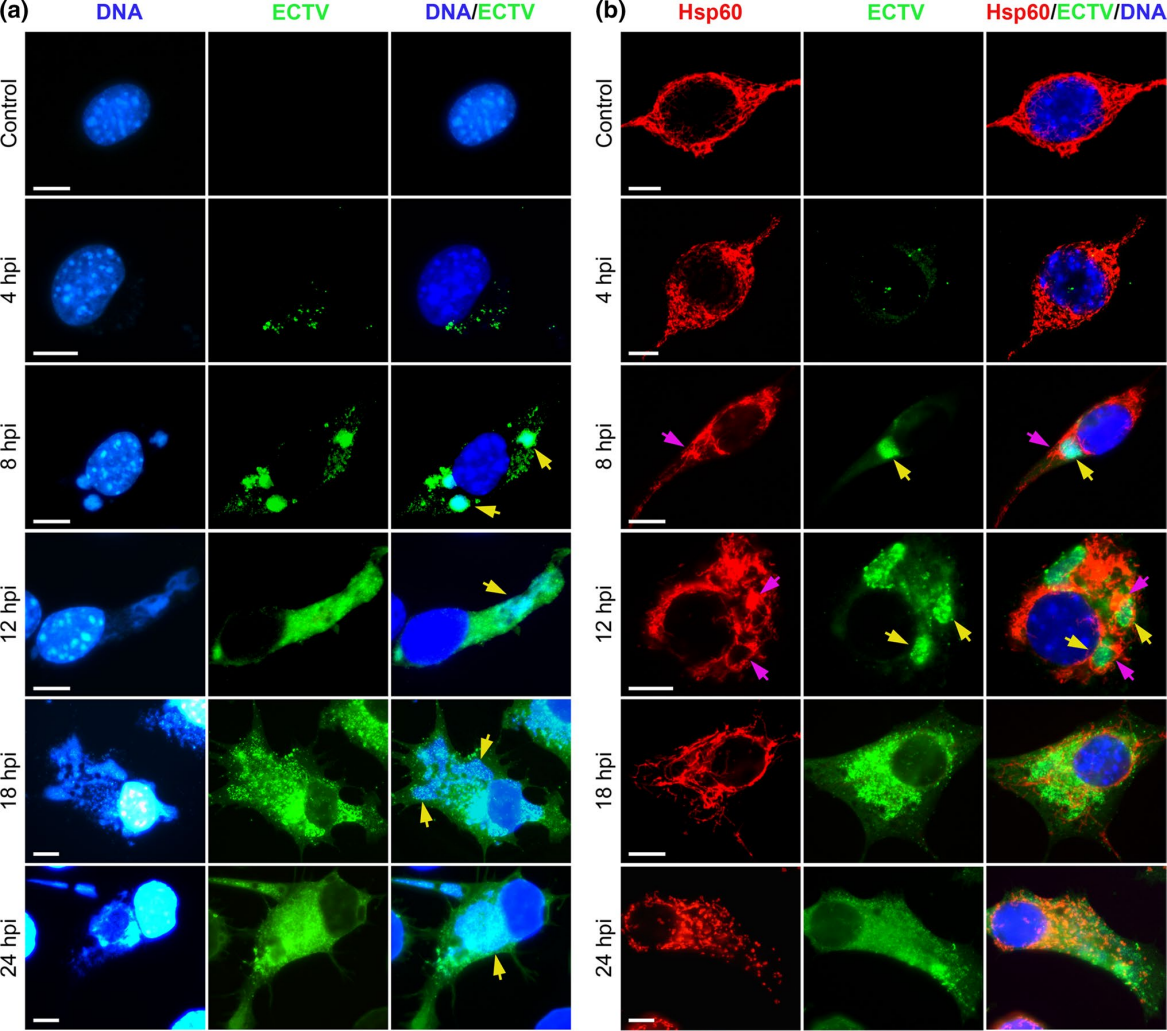
ECTV replication in L929 cells and Hsp60 localization. **a** Representative images show the kinetic of ECTV replication cycle in L929 fibroblasts. Green fluorescence—ECTV antigens; blue fluorescence—nuclear and/or viral DNA. **b** Representative images of Hsp60 localization in ECTV-infected cells at 4, 8, 12, 18, and 24 hpi. Red fluorescence—Hsp60; green fluorescence—ECTV antigens; blue fluorescence—nuclear and/or viral DNA. Arrows indicate viral factories (yellow) or clustering of Hsp60 around viral factories (purple). Scale bars: 20 µm. Fluorescence microscopy images are representative of one of five independent biological replicates. The number of cells evaluated was at least 50 for each condition per individual experiment

Initially, we investigated whether the replication of ECTV in permissive L929 fibroblasts influenced intracellular distribution of Hsp60. At 4 hpi, the majority of fibroblasts exhibited the presence of single virions on/inside the cells, without formation of viral factories (viroplasms) (Fig. 1a). At this time point, no differences in Hsp60 localization were detected compared to control cells, and its distribution was mostly perinuclear and probably reflected location of mitochondria (Fig. 1b). Between 8 and 12 hpi, regular viral factories were clearly visible within the cytoplasm of infected cells, and Hsp60 started to surround sites of viral replication (Fig. 1b). Between 18 and 24 hpi, when viral factories acquired the bloated form, distribution of Hsp60 changed from perinuclear to diffuse throughout the cytoplasm (Fig. 1b).

### Localization of Hsp60 Remains Mitochondrial During ECTV Life Cycle in L929 Fibroblasts

**Fig. 2:**
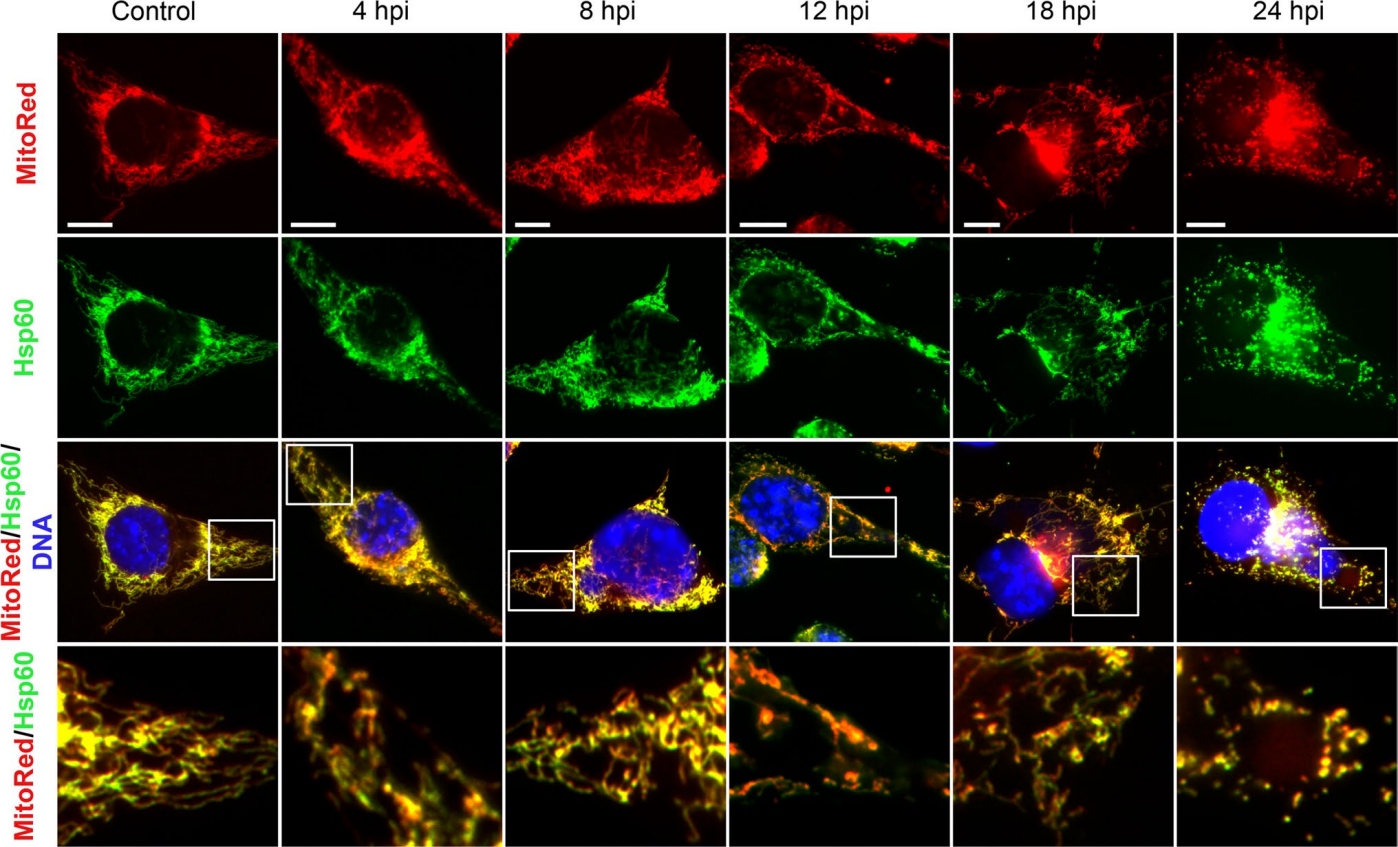
Hsp60 localization in L929 cells during ECTV replication cycle. Red fluorescence—mitochondria; green fluorescence—Hsp60; blue fluorescence—nuclear and/or viral DNA. The magnified images are of the boxed regions. Scale bars: 20 µm. Fluorescence microscopy images are representative of one of five independent biological replicates. The number of cells evaluated was at least 50 for each condition per individual experiment

**Fig. 3:**
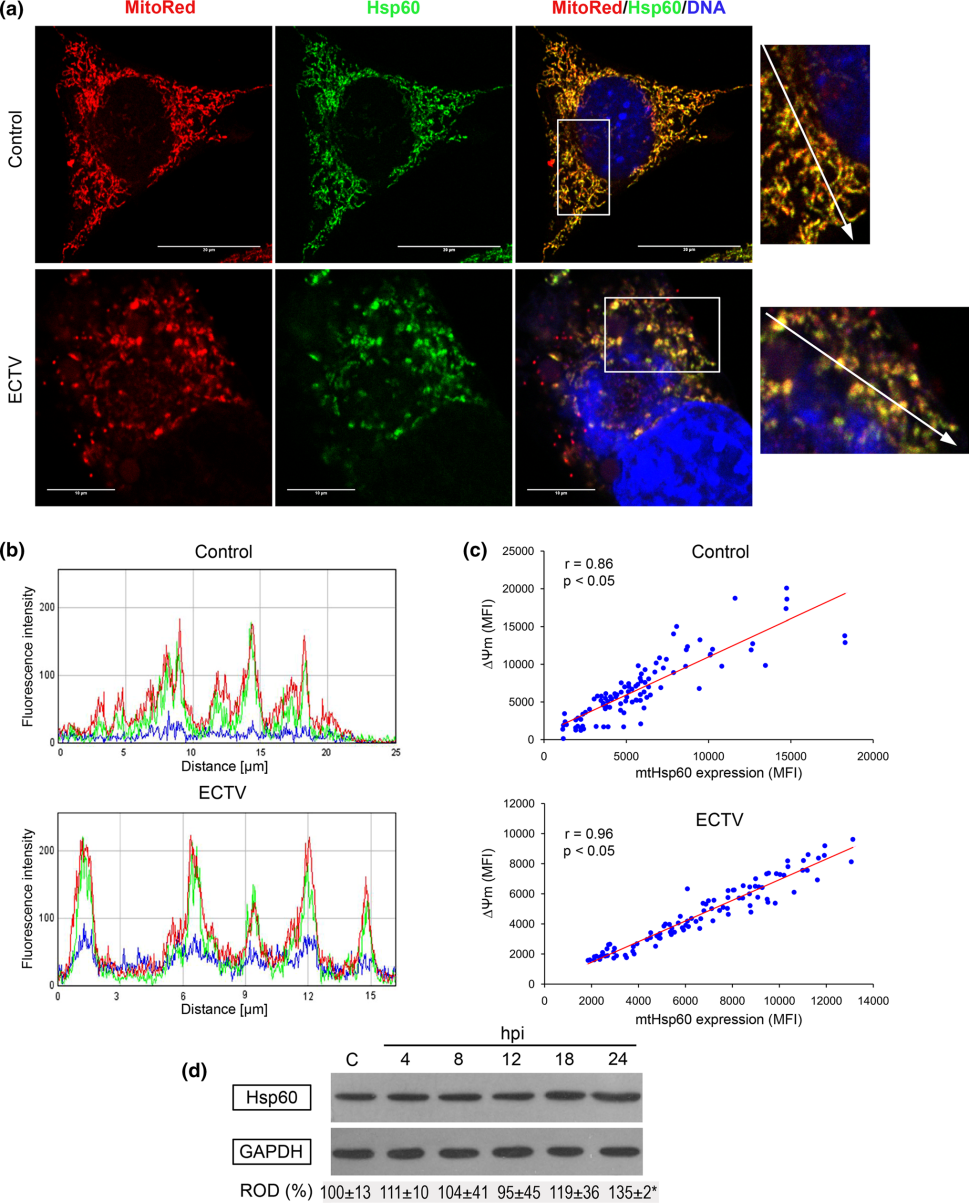
Hsp60 level correlates positively with mitochondrial membrane potential (Δψm) in ECTV-infected L929 cells. **a** Representative images show Hsp60 co-localization with mitochondria in control (upper panel) and ECTV-infected (lower panel) L929 cells at 24 hpi. Red fluorescence—mitochondria; green fluorescence—Hsp60; blue fluorescence—nuclear and/or viral DNA. The magnified images are of the boxed regions. Scale bars: 20 µm (upper panel) and 10 µm (lower panel). **b** Mean fluorescent intensity (MFI) of red (mitochondria), green (Hsp60), and blue (nuclear and/or viral DNA) fluorescence measured along the line marked by the white arrows in magnified images. **c** Correlation between Hsp60 level and mitochondrial potential (both expressed as MFI) in control and ECTV-infected cells at 24 hpi (Pearson correlation coefficient 0.94 and 0.96, respectively; *p < 0.05; *n *= 100). **d** Western blot analysis of Hsp60 level in control (C) and ECTV-infected L929 cells at 4, 8, 12, 18, and 24 hpi. Numbers represent mean ± SD of relative level of Hsp60 with respect to the control, which was considered as 100%. GAPDH was used as a loading control (Student’s *t* test; *p < 0.05). ROD: relative optical density. Confocal microscopy and western blot images are representatives of one of the three independent biological replicates. The number of cells evaluated by confocal microscopy was at least 50 for each condition per individual experiment. Quantitative data are expressed as mean ± standard deviation (SD)

Because distribution of Hsp60 in L929 cells seemed to be strictly mitochondria-dependent, we performed additional staining using membrane potential-dependent MitoRed dye. Indeed, in control and infected cells at all stages of virus replication (4–24 hpi), the chaperone strongly co-localized with mitochondria (Fig. 2). Confocal microscopy analysis (Fig. 3a) and evaluation of MFI of red (∆Ψm) and green (Hsp60) fluorescence (Fig. 3b) confirmed highly positive correlation between the level of Hsp60 and membrane potential of mitochondria in L929 cells (Fig. 3c). In uninfected cells, the Pearson correlation coefficient was 0.86 (*p* ≤ 0.05) for the two quantitative MFI measurements, whereas in infected cells at 24 hpi, it increased to 0.96 (*p* ≤ 0.05).

### The Level of Hsp60 Increases During Later Stages of ECTV Infection in L929 Cells

Next, we examined the influence of ECTV infection on the intracellular level of Hsp60 in murine fibroblasts. During the first 12 hpi, Hsp60 level in infected cells did not change in comparison to the control. The level of chaperone started to increase from 18 hpi, and at 24 hpi, it was significantly (*p* ≤ 0.05) elevated comparing to the level observed in uninfected cells (Fig. 3d).

### ECTV Infection of L929 Fibroblasts Increases Intracellular Level of Hsp10 and Its Co-localization with Mitochondria

**Fig. 4:**
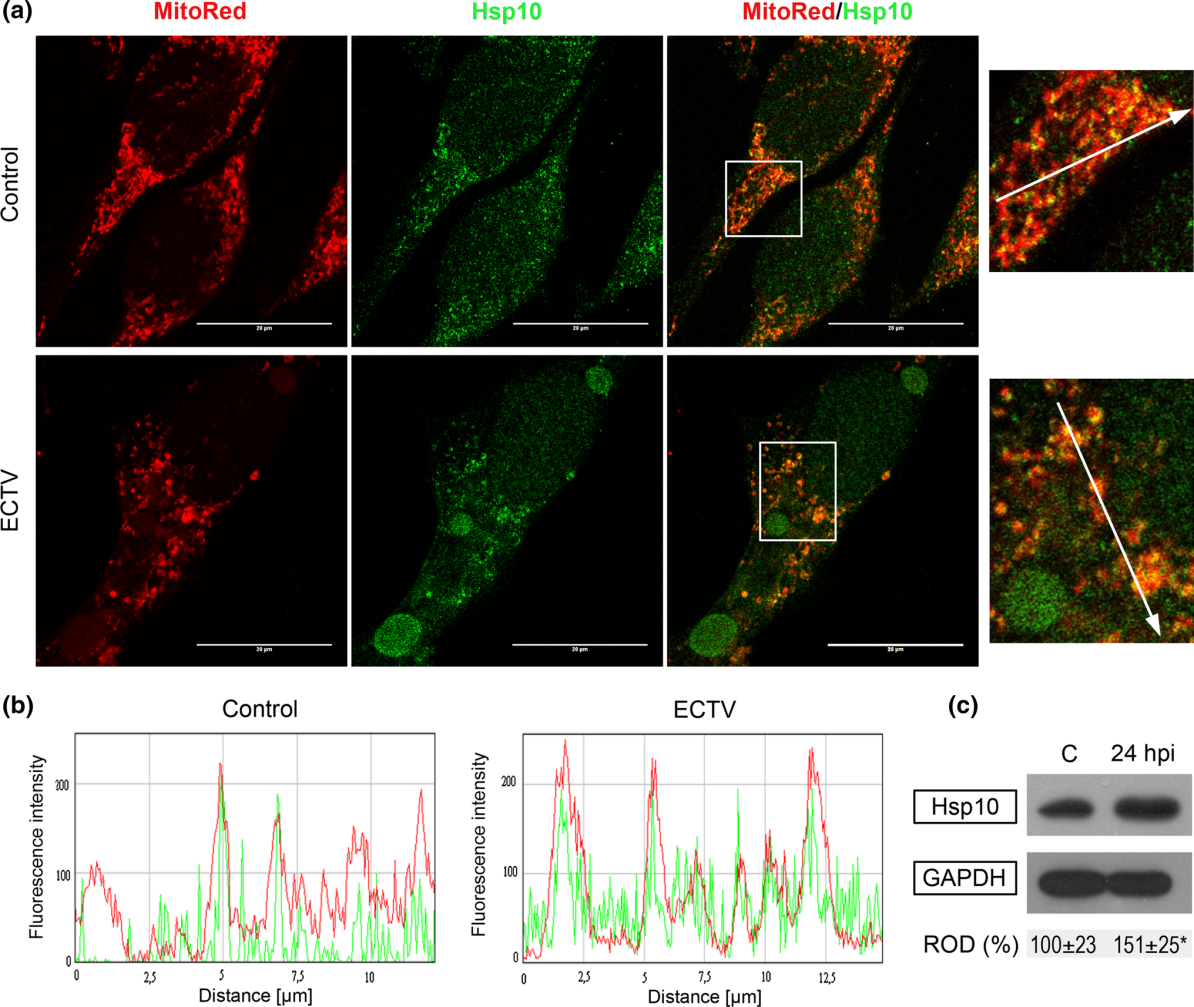
Level of Hsp10 and its localization in control and ECTV-infected L929 fibroblasts. **a** Representative images show Hsp10 distribution in control (upper panel) and ECTV-infected (lower panel) L929 cells at 24 hpi. Red fluorescence—mitochondria; green fluorescence—Hsp10. The magnified images are of the boxed regions. Scale bars: 20 µm. **b** Mean fluorescent intensity (MFI) of red (mitochondria) and green (Hsp10) fluorescence measured along the line marked by the white arrows in magnified images. **c** Western blot analysis of Hsp10 level in control (C) and ECTV-infected L929 cells at 24 hpi. Numbers represent mean ± SD of relative level of Hsp10 with respect to the control, which was considered as 100%. GAPDH was used as a loading control (Student’s *t* test; *p < 0.05). ROD: relative optical density. Confocal microscopy and western blot images are representatives of one of three independent biological replicates. The number of cells evaluated by confocal microscopy was at least 50 for each condition per individual experiment. Quantitative data are expressed as mean ± standard deviation (SD)

Hsp60 performs its chaperone functions with its cofactor, Hsp10. Therefore, we used double fluorescence staining of mitochondria and Hsp10 coupled with confocal microscopy analysis to visualize intracellular Hsp10 distribution. In both control and infected cells at 24 hpi, Hsp10 was mainly localized in mitochondria, but it was also observed in other subcellular compartments, such as nucleus and cytosol (Fig. 4a). However, in ECTV-infected cells, increased co-localization between Hsp10 and mitochondrial network was observed (Fig. 4b). In addition, western blot analysis revealed significant (*p* ≤ 0.05) increase in Hsp10 level in infected cells at 24 hpi, compared to control cells (Fig. 4c).

### The Effect of ECTV Infection on Mitochondrial Mass and Selected Subunits of ETC Complexes

**Fig. 5:**
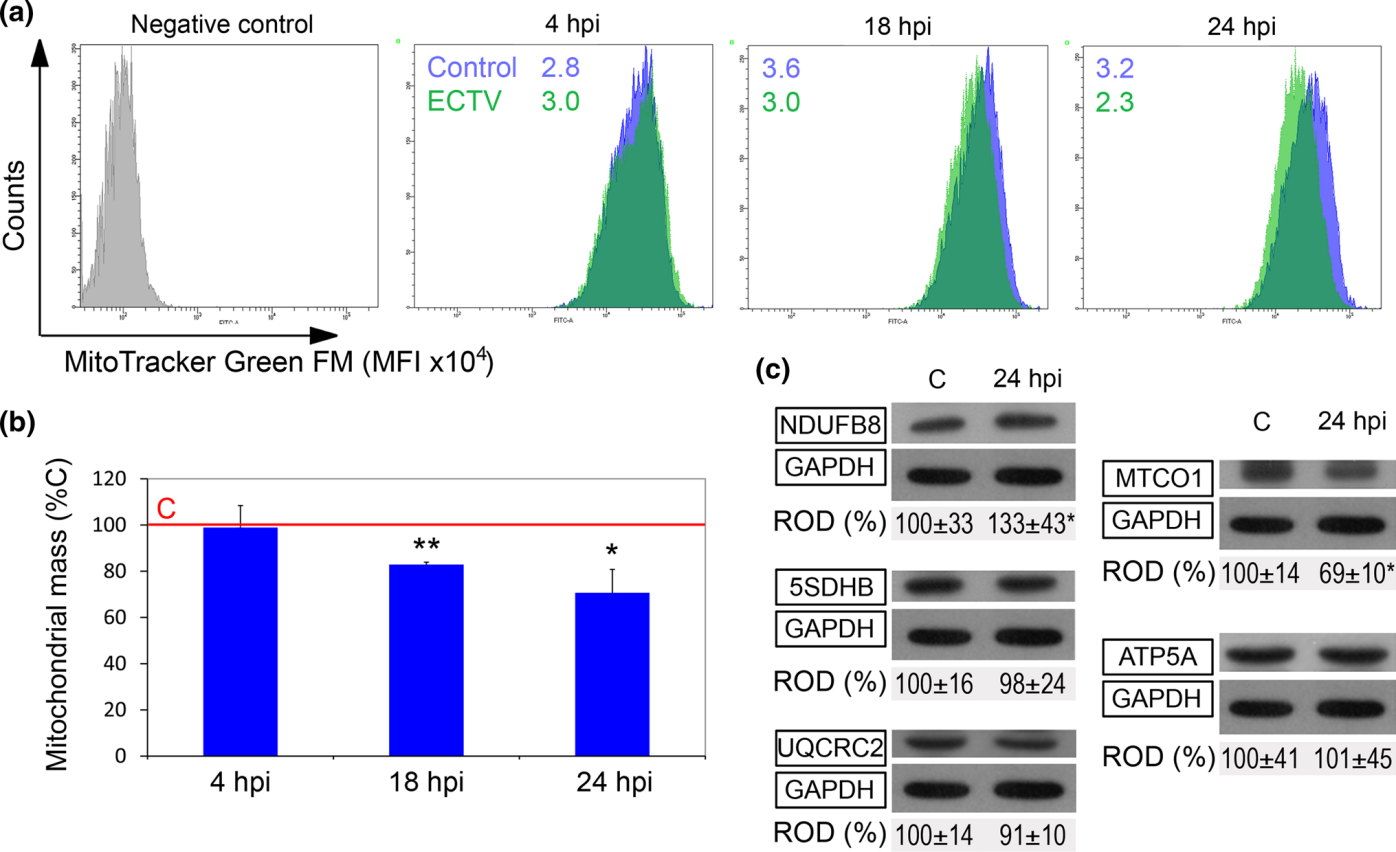
ECTV decreases mitochondrial mass in L929 fibroblasts at later stages of infection. **a** Representative histograms show MFI of MitoTracker Green FM in control (blue) and ECTV-infected (green) L929 cells at 4, 18, and 24 hpi. Unstained cells were used as a negative control (gray). **b** Relative mitochondrial mass with respect to the control (C) which was considered as 100%; data show mean ± SD (paired Student’s *t* test; *p < 0.05, **p < 0.01). **c** Western blot analysis of the level of selected subunits of electron transport chain complexes. Numbers represent mean ± SD of relative level of selected subunits of electron transport chain complexes with respect to the control, which was considered as 100%. GAPDH was used as a loading control (Student’s *t* test; *p < 0.05). ROD: relative optical density. Flow cytometry and western blot images are representatives of one of three independent biological replicates. Quantitative data are expressed as mean ± standard deviation (SD)

Because mtHsps are important for maintaining mitochondrial biogenesis, we determined the effect of ECTV infection on mitochondrial mass, which is a surrogate marker for biogenesis of mitochondria. The amounts of mitochondria were assessed using MitoTracker Green FM and subsequent flow cytometry analysis. At 4 hpi, the mitochondrial mass remained unchanged compared to the control; however, at 18 and 24 hpi, it decreased significantly (p ≤ 0.05) by 18% and 29%, respectively, (Fig. 5a and b). Because mitochondrial mass determines mitochondrial ATP production, which in turn relies on the ETC, next, we evaluated the level of selected respiratory chain complexes (NDUFB8, 5SDHB, UQCRC2, and MTCO1) and a subunit of ATPase (ATP5A) in ECTV-infected cells at 24 hpi (Fig. 5c). The level of 5SDHB (complex II), UQCRC2 (complex III), and ATP5A (complex V) remained stable, whereas the amount of NDUFB8 (complex I) and MTCO1 (complex IV) increased or decreased significantly (*p* ≤ 0.05), respectively, in ECTV-infected cells compared to control values (Fig. 5c).

### ECTV Infection Does Not Induce Apoptosis in L929 Cells During the 24 h Replication Cycle

**Fig. 6:**
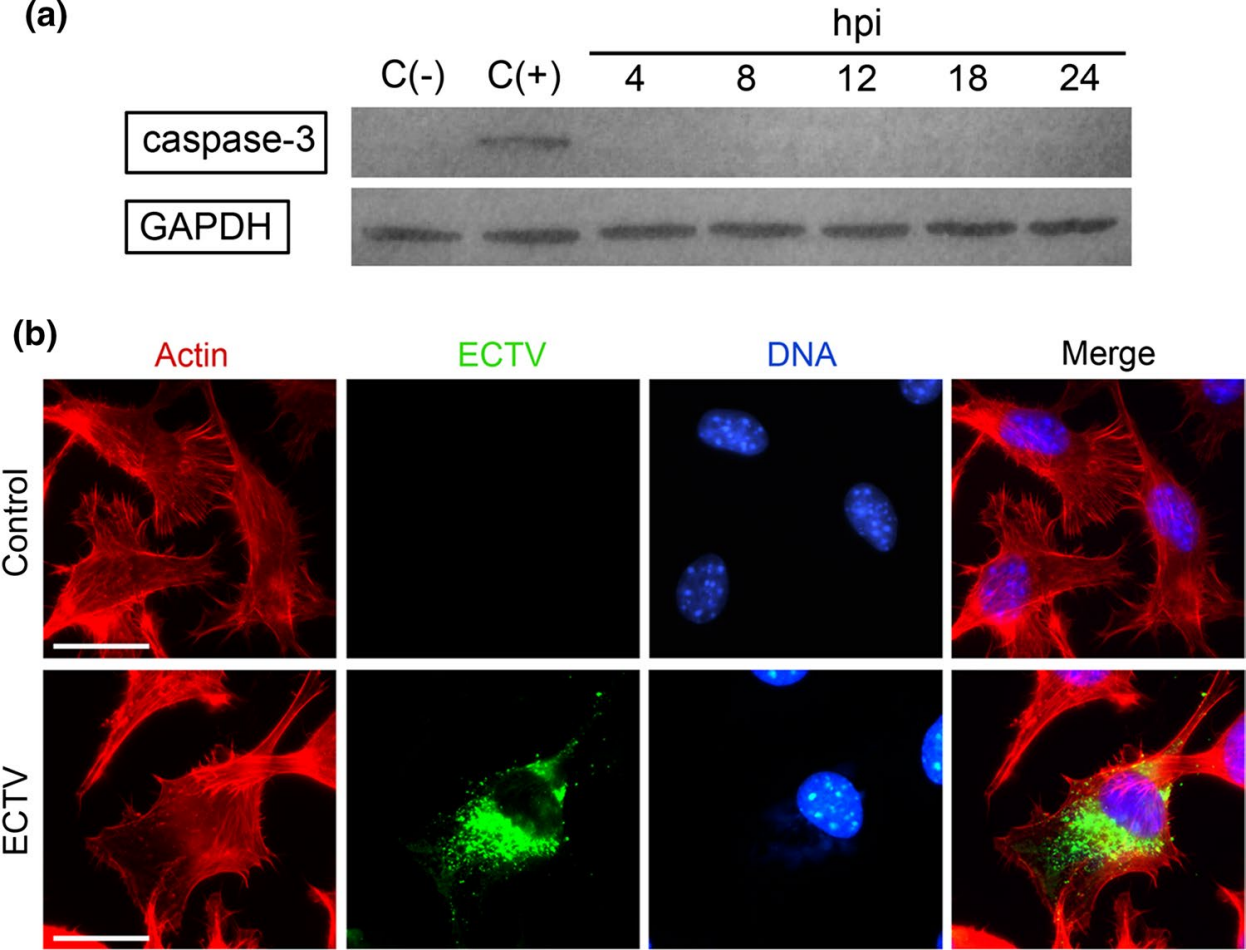
ECTV infection does not induce apoptosis in L929 fibroblasts. **a** Western blot analysis of the caspase-3 active form level in L929 cells during ECTV infection. C(–)—negative control (uninfected cells untreated with staurosporine); C(+)—positive control (uninfected cells treated with 2 µM staurosporine for 8 h). GAPDH was used as a loading control. **b** Representative images show morphology of control and ECTV-infected cells at 24 hpi. Red fluorescence—actin; green fluorescence—ECTV antigens; blue fluorescence—nuclear and/or viral DNA. Scale bars: 20 µm. Western blot and fluorescence microscopy images are representatives of one of three and seven, respectively, independent biological replicates

Mitochondrial localization of Hsp60 and increased level of mitochondrial chaperones during ECTV infection in L929 cells may suggest their mitoprotective role. Therefore, our last question concerned if apoptosis is executed in ECTV-infected cells, especially at the late stages of infection. Our previous study has shown that in contrast to RAW 264.7 macrophages, L929 fibroblasts do not show significant changes in the percentage of early or late apoptotic cells due to infection with ECTV, despite an increase in generation of reactive oxygen species (ROS), decrease in mitochondrial membrane potential and mitochondrial mass, and imbalance between mitochondrial fission and fusion (Gregorczyk et al. 2018). Lack of apoptosis induction was further confirmed by determination of caspase-3 activation using Western blot analysis, and unsurprisingly, the active form of the enzyme was not detected at any time point (4–24 hpi) (Fig. 6a). In addition, in ECTV-infected cells at 24 hpi, we did not observe any typical morphological changes characteristic for the apoptotic process, such as cell shrinkage and rounding, extensive plasma membrane blebbing, or nuclear defragmentation (Fig. 6b).

**Fig. 7:**
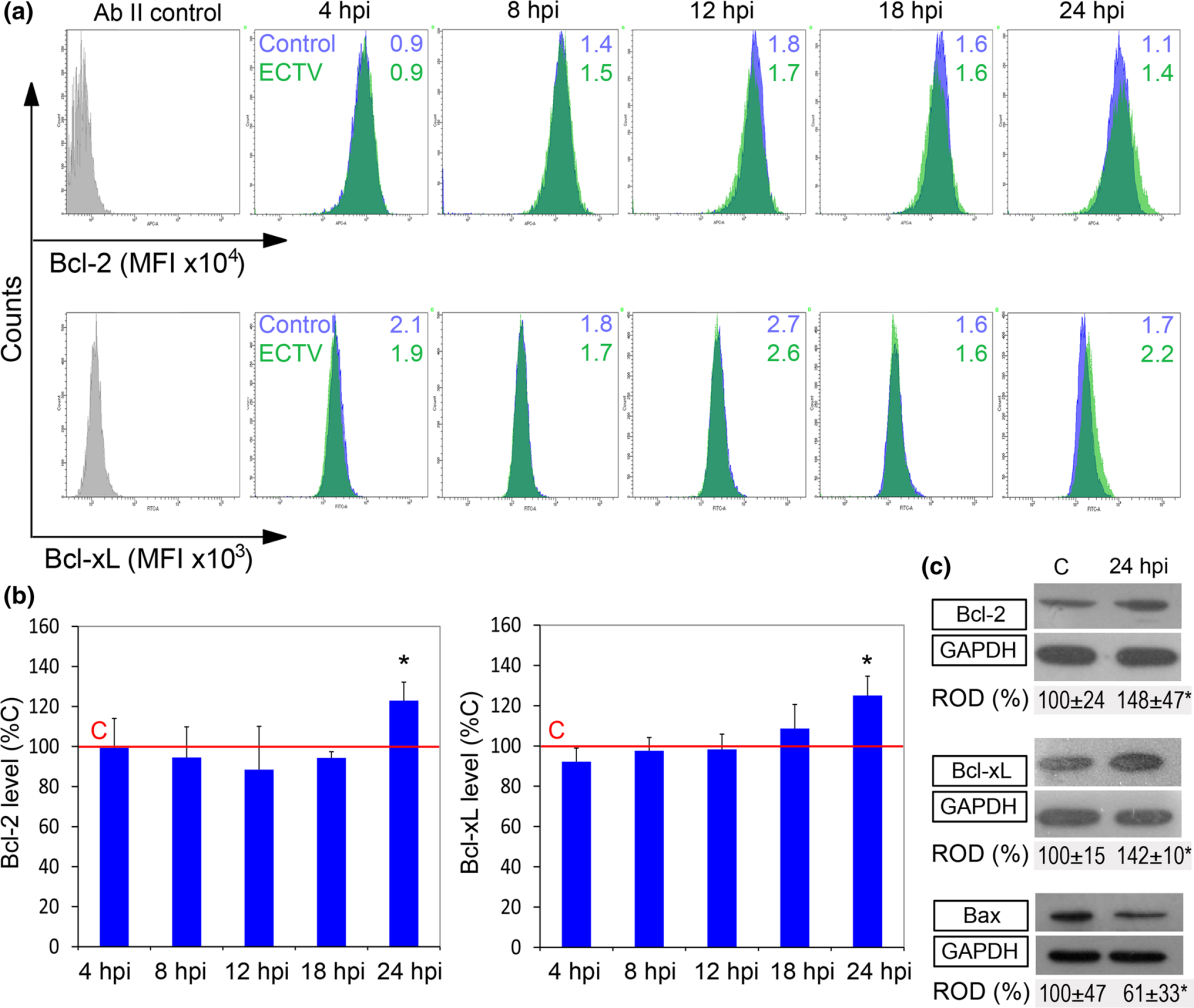
Changes in the expression of selected Bcl-2 family members in L929 fibroblasts during later stages of ECTV infection. **a** Representative histograms show mean fluorescent intensity (MFI) for Bcl-2 (upper panel) and Bcl-xL (lower panel) in control (blue) and ECTV-infected (green) L929 cells at 4, 8, 12, 18, and 24 hpi. Cells stained with secondary antibodies (Ab II) were used as a negative control (gray). **b** Graphs represent mean ± SD of relative Bcl-2 and Bcl-xL level with respect to the control (C), which was considered as 100% (paired Student’s *t* test; *p < 0.05). **c** Western blot analysis of the level of Bax, Bcl-2, and Bcl2-xL in control and ECTV-infected fibroblasts at 24 hpi. Numbers represent mean ± SD of relative level of proteins with respect to the control, which was considered as 100%. GAPDH was used as a loading control (Student’s *t* test; *p < 0.05). ROD: relative optical density. Flow cytometry and western blot images are representatives of one of three independent biological replicates. Quantitative data are expressed as mean ± standard deviation (SD)

Because induction of mitochondrial pathway of apoptosis is regulated by members of the Bcl-2 family, we measured the intracellular expression level of anti-apoptotic Bcl-2 and Bcl-xL, and pro-apoptotic Bax. Flow cytometry analysis revealed that between 4 and 18 hpi, the protein expression level of both, Bcl-2 and Bcl-xL, did not change significantly (*p* > 0.05) in infected cells compared to control cells (Fig. 7a and 7b). Meanwhile, at 24 hpi, the MFI of Bcl-2 and Bcl-xL increased significantly (*p* ≤ 0.05) by 22% and 25%, respectively, compared to control. Western blot analysis confirmed that in ECTV-infected cells at 24 hpi, the level of Bcl-2 and Bcl-xL significantly (*p* ≤ 0.05) increased compared to control values, by 48% and 42%, respectively, (Fig. 7c). Furthermore, the level of pro-apoptotic Bax protein was significantly (*p* ≤ 0.05) lower (39%) in ECTV-infected L929 cells at 24 hpi, compared to control cells (Fig. 7c). Taken together, our data indicate that ECTV infection in L929 cells increases anti-apoptotic potential of L929 cells, and therefore, the apoptosis is not induced but rather is inhibited.

## Discussion

Studies on molecular mechanisms of apoptotic regulation during viral infection may have potential therapeutic implications. The investigations should be performed to identify both viral and cellular proteins engaged in apoptosis control. It is possible that Hsp60 is one of the apoptotic regulators that affects cell viability and consequently influences the effectiveness of orthopoxvirus replication. Until now, many studies have confirmed the role of Hsp60 in apoptosis regulation in various cell types and pathologic conditions (Cohen-Sfady et al. 2009; Ghosh et al. 2008; Gruber et al. 2010; Resa-Infante et al. 2011). Hsp60 acts mainly as an anti-apoptotic protein that prevents apoptosis by maintaining mitochondrial integrity and stability, and by modifying the proportion between pro- and anti-apoptotic Bcl-2 family members (Ghosh et al. 2008; Gupta and Knowlton 2005; Shan et al. 2003). However, the importance of Hsp60 in the context of a poxviral infection has not been studied adequately.

In the present study, we used a highly virulent Moscow strain of ECTV and permissive L929 fibroblasts to elucidate the effect of a poxviral infection on the mitochondrial Hsps (Hsp60 and Hsp10). Our results showed that during the entire replication cycle of ECTV in L929 cells, the intracellular distribution of Hsp60 remained mostly mitochondrial. Our observation is consistent with a previous investigation showing the dominance of mitochondrial localization of Hsp60 over its cytoplasmic placement (Cechetto et al. 2000; Magen et al. 2008; Soltys and Gupta 1996). Using electron microscopy analysis, Soltys and Gupta (1996) confirmed that mitochondrial Hsp60 accounts for 80–85% of the total cellular Hsp60 in different mammalian cells and tissues, including human fibroblasts, Daudi Burkitt’s lymphoma cells, B-SC kidney cells, Chinese hamster ovary cells, and rat liver. It has been reported that mitochondrial localization of Hsp60 prevents apoptosis, whereas its release to the cytosol promotes apoptosis (Chandra et al. 2007; Samali et al. 1999). Hsp60 translocation from mitochondria to the cytosol leads to increase in the caspase-3 activation rate in human GM701 fibroblasts (Chandra et al. 2007). This result is consistent with the previous report of Samali et al. (1999) that release of mitochondrial Hsp60 favors the activation of the caspase-3. Hsp60 release to the cytosol depends on the pro-apoptotic Bax protein (Chandra et al. 2007), which conformational activation has been found to be inhibited by ECTV protein—EVM025—resulting in prevention of apoptosis (Mehta et al. 2015). Therefore, it is not excluded that mitochondrial localization of Hsp60 observed in ECTV-infected L929 cells allows maintenance of their anti-apoptotic state.

Our study also revealed that ECTV increases the level of Hsp60 and Hsp10 in fibroblasts during later stages of infection. The intensification of Hsp60 expression may occur in response to various stressors, as well as pathological states, and is responsible for maintaining protein homeostasis within the cytosol and mitochondria (Chandra et al. 2007; Itoh et al. 2002). Increase in Hsp60 level can be the cell reaction for stress factors, such as heat shock (Mahanty et al. 2016), dehydration (Itoh et al. 2002), heavy metals (Rios-Arana et al. 2005), and oxidative stress caused by hyperglycemia (Hall and Martinus 2013). Intensified Hsp60 synthesis is observed in diseases associated with excessive ROS production, such as temporal lobe epilepsy (Gammazza et al. 2015), Crohn’s disease, and ulcerative colitis (Alzoghaibi 2013; Rodolico et al. 2010; Wang et al. 2016). A previous study indicates that ROS, especially H_2_O_2_, may be involved in the transduction of the signal inducing transcription of Hsps genes, including Hsp60-encoding gene (Hall and Martinus 2013). H_2_O_2_ enhances heat shock factor (HSF)-1 DNA-binding activity by oxidizing its two cysteine residues and consequently causing disulfide bridge formation in DNA-binding domain of HSF-1. H_2_O_2_ also promotes HSF-1 translocation to the cell nucleus (Ahn and Thiele 2003). In the meantime, our previous reports have demonstrated that at later stages (18–24 hpi) of infection with ECTV, the formation of donut-like and discrete mitochondria, as well as increase in ROS generation are observed in L929 cells (Gregorczyk et al. 2014b, 2018). Altered fusion dynamics and formation of donut-shaped mitochondria occur in response to hypoxia and those structures are known as the main source of ROS (Liu and Hajnóczky 2011). In ECTV-infected cells, the generation of donut-like and discrete mitochondria coincides with increase in intracellular level of Hsp60, and therefore, we hypothesize that elevation of Hsp60 level can be induced by ROS. In a positive feedback loop, overexpression of Hsp60 may in turn prevent further increase in ROS to a level that does not induce cell apoptosis. Kang et al. (2009) have shown that hepatitis C virus (HCV) core protein-dependent inhibition of the Hsp60 activity leads to ROS increase and, consequently, raises the rate of tumor necrosis factor-α-induced apoptosis in HCV-infected Huh7 and Huh7TR cells. Kleinridders et al. (2013) observed that Hsp60 gene inactivation disrupts mitochondrial functionality and increases ROS production in N25/2 mouse cells. Therefore, the mitochondrial chaperone Hsp60 may counteract excessive ROS production and ROS-dependent induction of mitochondrial pathway of apoptosis. (Lin et al. 2001; Tang et al. 2016).

Hsp60 performs its chaperone functions with its cofactor, Hsp10. In ECTV-infected cells, Hsp10 was localized within mitochondria, cytosol, and the nucleus, what are consistent with the report by Corrao et al. (2014) who performed the study on human lung fibroblasts HFL-1. The mitochondrial localization of Hsp10 was intensified during later stages of ECTV infection, suggesting anti-apoptotic and mitoprotective Hsp10 activity. Additionally, Hsp10 level also increased in ECTV-infected fibroblasts. Simultaneous increase in quantity of both chaperones may be the result of coincidental expression of their genes. Human genome analysis revealed that *hspe1* and *hspd1* genes encoding Hsp10 and Hsp60, respectively, have common bidirectional promoter, as well as they are controlled by the same regulatory mechanisms (Hansen et al. 2003). Simultaneous increase in both Hsp60 and Hsp10 expressions is also observed in various types of mammalian tissues and cells during pathological conditions, such as myocardial ischemia (Lau et al. 1997; Lin et al. 2001) or cancer (Cappello et al. 2003, 2008). It has been shown that overexpression of Hsp60 and Hsp10 individually or in combination with one another protects cardiac myocytes against apoptotic cell death induced by hypoxia reoxygenation. Individually, Hsp60 had a greater anti-apoptotic potential than Hsp10 (Shan et al. 2003).

Hsp60 is responsible for maintaining of mitochondrial functionality and for promoting ETC activity. Lin et al. (2001) reported that overproduction of Hsp60 (alone or together with Hsp10) is accompanied with the higher activity of ETC complexes III and IV. Complex I, III, and V activity disruption may cause intensification in ROS production (Sipos et al. 2003; Yoon et al. 2005). Our results show that ECTV infection only moderately influences expression of subunits of the respiratory chain; therefore, we cannot exclude that increase in Hsp60/Hsp10 may be part of the mechanism that counteracts oxidative stress by chaperoning mitochondrial proteins, including ETC complex subunits. However, this statement should be qualified with further investigations.

Numerous reports confirm the role of Hsp60 (alone or in cooperation with Hsp10) in apoptosis regulation (Cohen-Sfady et al. 2009; Ghosh et al. 2008; Gruber et al. 2010; Resa-Infante et al. 2011; Shan et al. 2003). This appears to support the notion that Hsp60 influences the effectiveness of ECTV replication by preventing apoptosis of the host cells. Indeed, increased expression of Hsp60 and Hsp10 was accompanied by decreased apoptotic potential of L929 cells. The quantitative relationship between the Bcl-2 family proteins changed in the way that increased resistance of ECTV-infected cells to apoptotic stimulus, thus promoting their survival. Additionally, the persistence of ECTV infection occurring in immune cells of susceptible mouse strains (Sakala et al. 2015; Spohr de Faundez et al. 1995) provokes the question about mechanisms responsible for maintaining infected cell viability. Shan et al. (2003) observed that Hsp60 and Hsp10 increased the level of Bcl-2 and Bcl-xL, but decreased the level of Bax in the rat cardiomyocytes. Moreover, overproduction of Hsp60 or Hsp10 enhanced cell resistance to oxidative stress induced by doxorubicin (Shan et al. 2003). Additionally, Kirchhoff et al. (2002) determined a negative correlation between Hsp60 level and quantity of Bax and Bak, and observed positive dependence between intracellular Hsp60 and Bcl-2. Both reports are consistent with our results showing that in L929, fibroblasts at the late stage of ECTV infection (24 hpi) increase the expression of Bcl-2 and Bcl-xL, but decrease expression of Bax with concurrent high expression of Hsp60 and Hsp10. Therefore, we cannot exclude that Hsp60 and/or Hsp10 may contribute to the lowering of cell apoptotic potential by modifying the proportion of Bax, Bcl-2, and Bcl-xl-L proteins. However, this statement requires further investigations using experiments with silencing or removing the Hsp60 gene or by specific protein inhibitors.

The molecular mechanism underlying the interactions between Hsp60/Hsp10 and Bcl-2 family proteins is not fully understood; however, probably Hsp60 regulates post-translational modifications of these proteins rather than influences their mRNA level (Kirchhoff et al. 2002; Shan et al. 2003). It has been revealed that Hsp60 inhibits Bcl-2 degradation (Kirchhoff et al. 2002) and makes complexes with Bcl-xL preventing its ubiquitination and probably subsequent proteasomal degradation (Shan et al. 2003) in cardiac myocytes. Hsp60 is also able to bind Bax and presumably decrease Bax level by the post-translational interaction accelerating Bax degradation (Shan et al. 2003). On the other hand, both cytosolic and mitochondrial Hsp60 are able to bind p53 protein and decrease its stability in HCT116 cells. p53 positively regulates the gene-encoding Bax (Ghosh et al. 2008) as well as down-regulates Bcl-2-encoding gene transcription (Miyashita et al. 1994).

Taken together, our results show for the first time that ECTV infection upregulates the expressions of Hsp60 and Hsp10, and decreases apoptotic potential of L929 fibroblasts during later stages of infection. We hypothesize that Hsp60 and/or its cofactor counteract induction of the intrinsic pathway of apoptosis by maintaining protein homeostasis in mitochondria contributing to change in Bax, Bcl-2, and Bcl-xL ratio. The question whether Hsp60 could serve as a novel therapeutic target for the treatment of orthopoxviral diseases remains open and requires further investigation.
